# 7,11,18,21-Tetra­oxatrispiro­[5.2.2.5.2.2]heneicosa­ne

**DOI:** 10.1107/S1600536809040884

**Published:** 2009-10-10

**Authors:** Jiang-Hua Shi, Xian-You Yuan, Min Zhang, Seik Weng Ng

**Affiliations:** a College of Chemistry, Xiangtan University, Xiangtan 411105, People’s Republic of China; b Department of Biology and Chemistry, Hunan University of Science and Engineering, Yongzhou, Hunan 425100, People’s Republic of China; c Department of Chemistry, University of Malaya, 50603 Kuala Lumpur, Malaysia

## Abstract

The four six-membered rings all adopt chair conformations in the two independent mol­ecules of the title cyclo­hexa­none cyclic diacetal with penta­erythritol, C_17_H_28_O_4_.

## Related literature

For low-temperature manifestation of chirality as evidenced by solution NMR, see: Dodziuk *et al.* (1991 ▶). For the crystal structure of 6,10,16,19-tetra­oxatrispiro­[4.2.2.4.2.2]nona­decane, see: Wang *et al.* (2008 ▶).
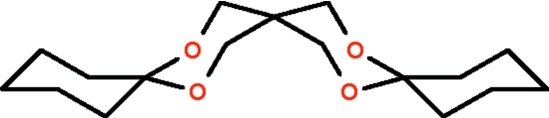


## Experimental

### 

#### Crystal data

C_17_H_28_O_4_
*M* *_r_* = 296.39
Monoclinic, 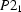

*a* = 11.1256 (5) Å
*b* = 13.9106 (7) Å
*c* = 11.6500 (6) Å
β = 118.002 (1)°
*V* = 1591.92 (14) Å^3^
*Z* = 4
Mo *K*α radiation
μ = 0.09 mm^−1^
*T* = 293 K
0.48 × 0.42 × 0.26 mm

#### Data collection

Bruker SMART diffractometer
Absorption correction: none
11846 measured reflections
3620 independent reflections
3268 reflections with *I* > 2σ(*I*)
*R*_int_ = 0.031

#### Refinement

*R*[*F* ^2^ > 2σ(*F* ^2^)] = 0.039
*wR*(*F* ^2^) = 0.110
*S* = 1.01
3620 reflections
380 parameters
1 restraint
H-atom parameters constrained
Δρ_max_ = 0.23 e Å^−3^
Δρ_min_ = −0.22 e Å^−3^

### 

Data collection: *SMART* (Bruker, 1997 ▶); cell refinement: *SAINT* (Bruker, 2003 ▶); data reduction: *SAINT*; program(s) used to solve structure: *SHELXS97* (Sheldrick, 2008 ▶); program(s) used to refine structure: *SHELXL97* (Sheldrick, 2008 ▶); molecular graphics: *X-SEED* (Barbour, 2001 ▶); software used to prepare material for publication: *publCIF* (Westrip, 2009 ▶).

## Supplementary Material

Crystal structure: contains datablocks global, I. DOI: 10.1107/S1600536809040884/tk2552sup1.cif

Structure factors: contains datablocks I. DOI: 10.1107/S1600536809040884/tk2552Isup2.hkl

Additional supplementary materials:  crystallographic information; 3D view; checkCIF report

## Figures and Tables

**Fig. 1.**
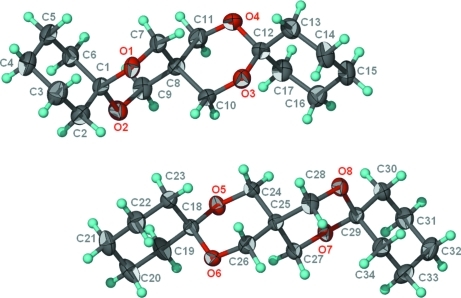
Thermal ellipsoid plot (Barbour, 2001) of the two independent molecules of C17H28O4 at the 50% probability level; hydrogen atoms are drawn as spheres of arbitrary radius.

## supplementary crystallographic information

### Experimental

Pentaerythritol (2 g, 0.014 mol), cyclohexanone (3.2 g, 0.033 mol), toluene (12 ml) and a catalytic amount (0.2 g) of *p*-toluenesulfonic acid were heated for four hours. The mixture was cooled and then filtered. The organic phase was washed with water and 5% sodium bicarbonate (20 ml). The solvent was evaporated and the product recrystallized from ethanol to afford colorless crystals (yield 60%); m.p. 386–387 K.

### Refinement

Carbon-bound H-atoms were placed in calculated positions (C–H 0.97 Å) and were included in the refinement in the riding model approximation, with *U*_iso_(H) set to 1.2*U*_eq_(C). In the absence of significant anomalous scattering effects, 3068 Friedel pairs were averaged in the final refinement.

### Crystal data

**Table:**

C_17_H_28_O_4_	*F*(000) = 648
*M**_r_* = 296.39	*D*_x_ = 1.237 Mg m^−^^3^
Monoclinic, *P*2_1_	Mo *K*α radiation, λ = 0.71073 Å
Hall symbol: P 2yb	Cell parameters from 6307 reflections
*a* = 11.1256 (5) Å	θ = 2.1–27.1°
*b* = 13.9106 (7) Å	µ = 0.09 mm^−^^1^
*c* = 11.6500 (6) Å	*T* = 293 K
β = 118.002 (1)°	Block, colorless
*V* = 1591.92 (14) Å^3^	0.48 × 0.42 × 0.26 mm
*Z* = 4	

### Data collection

**Table:**

Bruker SMART diffractometer	3268 reflections with *I* > 2σ(*I*)
Radiation source: fine-focus sealed tube	*R*_int_ = 0.031
graphite	θ_max_ = 27.1°, θ_min_ = 2.1°
φ and ω scans	*h* = −14→14
11846 measured reflections	*k* = −17→17
3620 independent reflections	*l* = −13→14

### Refinement

**Table:**

Refinement on *F*^2^	Hydrogen site location: inferred from neighbouring sites
Least-squares matrix: full	H-atom parameters constrained
*R*[*F*^2^ > 2σ(*F*^2^)] = 0.039	*w* = 1/[σ^2^(*F*_o_^2^) + (0.0742*P*)^2^ + 0.1289*P*] where *P* = (*F*_o_^2^ + 2*F*_c_^2^)/3
*wR*(*F*^2^) = 0.110	(Δ/σ)_max_ = 0.001
*S* = 1.01	Δρ_max_ = 0.23 e Å^−^^3^
3620 reflections	Δρ_min_ = −0.22 e Å^−^^3^
380 parameters	Extinction correction: *SHELXL97* (Sheldrick, 2008), Fc^*^=kFc[1+0.001xFc^2^λ^3^/sin(2θ)]^-1/4^
1 restraint	Extinction coefficient: 0.118 (7)
Primary atom site location: structure-invariant direct methods	Absolute structure: nd
Secondary atom site location: difference Fourier map	

### Fractional atomic coordinates and isotropic or equivalent isotropic displacement parameters (Å^2^)

**Table:**

	*x*	*y*	*z*	*U*_iso_*/*U*_eq_	
O1	0.23696 (16)	0.49997 (11)	0.73537 (14)	0.0470 (4)	
O2	0.42168 (15)	0.47553 (13)	0.93942 (16)	0.0553 (4)	
O3	0.25850 (16)	0.23362 (13)	0.67497 (16)	0.0512 (4)	
O4	0.10089 (15)	0.21168 (13)	0.75154 (18)	0.0570 (4)	
O5	0.69505 (15)	0.27388 (10)	0.85086 (14)	0.0425 (3)	
O6	0.83677 (14)	0.33303 (11)	0.76920 (15)	0.0422 (3)	
O7	0.75739 (13)	0.02803 (10)	0.76038 (13)	0.0395 (3)	
O8	0.56565 (13)	0.04986 (11)	0.55912 (14)	0.0450 (4)	
C1	0.3257 (2)	0.54471 (16)	0.8557 (2)	0.0442 (5)	
C2	0.4058 (2)	0.61848 (19)	0.8249 (3)	0.0575 (6)	
H2A	0.4468	0.5878	0.7769	0.069*	
H2B	0.4787	0.6430	0.9055	0.069*	
C3	0.3178 (3)	0.7017 (2)	0.7456 (3)	0.0658 (7)	
H3A	0.2521	0.6786	0.6605	0.079*	
H3B	0.3747	0.7493	0.7336	0.079*	
C4	0.2428 (3)	0.7485 (2)	0.8119 (3)	0.0658 (7)	
H4A	0.3079	0.7780	0.8928	0.079*	
H4B	0.1828	0.7985	0.7563	0.079*	
C5	0.1607 (3)	0.6742 (2)	0.8389 (3)	0.0612 (6)	
H5A	0.0903	0.6491	0.7572	0.073*	
H5B	0.1164	0.7043	0.8843	0.073*	
C6	0.2487 (2)	0.59221 (18)	0.9201 (2)	0.0516 (5)	
H6A	0.1919	0.5447	0.9323	0.062*	
H6B	0.3135	0.6163	1.0050	0.062*	
C7	0.1655 (2)	0.41903 (17)	0.7462 (2)	0.0486 (5)	
H7A	0.1130	0.3904	0.6610	0.058*	
H7B	0.1024	0.4399	0.7769	0.058*	
C8	0.2615 (2)	0.34349 (16)	0.8395 (2)	0.0430 (5)	
C9	0.3594 (3)	0.39432 (18)	0.9644 (2)	0.0560 (6)	
H9A	0.3104	0.4151	1.0102	0.067*	
H9B	0.4295	0.3495	1.0199	0.067*	
C10	0.3419 (2)	0.29263 (18)	0.7810 (2)	0.0487 (5)	
H10A	0.3856	0.3404	0.7524	0.058*	
H10B	0.4126	0.2537	0.8475	0.058*	
C11	0.1797 (3)	0.26731 (19)	0.8654 (2)	0.0555 (6)	
H11A	0.2410	0.2254	0.9347	0.067*	
H11B	0.1197	0.2980	0.8935	0.067*	
C12	0.1781 (2)	0.16498 (17)	0.7002 (2)	0.0464 (5)	
C13	0.0764 (2)	0.1282 (2)	0.5673 (3)	0.0613 (6)	
H13A	0.0317	0.1825	0.5110	0.074*	
H13B	0.0073	0.0907	0.5754	0.074*	
C14	0.1431 (3)	0.0666 (2)	0.5054 (3)	0.0661 (7)	
H14A	0.0735	0.0419	0.4231	0.079*	
H14B	0.2044	0.1062	0.4879	0.079*	
C15	0.2212 (3)	−0.0162 (2)	0.5910 (3)	0.0666 (7)	
H15A	0.2670	−0.0512	0.5507	0.080*	
H15B	0.1587	−0.0599	0.6008	0.080*	
C16	0.3257 (3)	0.0201 (2)	0.7239 (3)	0.0648 (7)	
H16A	0.3704	−0.0344	0.7797	0.078*	
H16B	0.3945	0.0572	0.7147	0.078*	
C17	0.2613 (3)	0.0820 (2)	0.7872 (2)	0.0567 (6)	
H17A	0.3323	0.1078	0.8679	0.068*	
H17B	0.2026	0.0423	0.8081	0.068*	
C18	0.75704 (19)	0.35744 (14)	0.83150 (18)	0.0361 (4)	
C19	0.8575 (3)	0.39214 (17)	0.9659 (2)	0.0539 (6)	
H19A	0.9274	0.3437	1.0079	0.065*	
H19B	0.8106	0.4004	1.0175	0.065*	
C20	0.9240 (3)	0.4863 (2)	0.9620 (2)	0.0642 (7)	
H20A	0.9829	0.5078	1.0501	0.077*	
H20B	0.9799	0.4763	0.9194	0.077*	
C21	0.8190 (3)	0.56338 (17)	0.8899 (3)	0.0608 (6)	
H21A	0.8645	0.6214	0.8846	0.073*	
H21B	0.7693	0.5784	0.9374	0.073*	
C22	0.7201 (3)	0.52960 (17)	0.7538 (2)	0.0544 (6)	
H22A	0.7686	0.5208	0.7038	0.065*	
H22B	0.6509	0.5784	0.7109	0.065*	
C23	0.6521 (2)	0.43532 (15)	0.7575 (2)	0.0447 (5)	
H23A	0.5940	0.4137	0.6693	0.054*	
H23B	0.5953	0.4459	0.7989	0.054*	
C24	0.61688 (19)	0.21936 (15)	0.7364 (2)	0.0396 (4)	
H24A	0.5395	0.2571	0.6765	0.047*	
H24B	0.5825	0.1621	0.7587	0.047*	
C25	0.70167 (19)	0.19016 (14)	0.67041 (18)	0.0351 (4)	
C26	0.7655 (2)	0.28130 (15)	0.6507 (2)	0.0425 (4)	
H26A	0.8280	0.2645	0.6175	0.051*	
H26B	0.6948	0.3218	0.5868	0.051*	
C27	0.81339 (19)	0.11917 (14)	0.7551 (2)	0.0413 (4)	
H27A	0.8764	0.1111	0.7200	0.050*	
H27B	0.8637	0.1449	0.8424	0.050*	
C28	0.6117 (2)	0.14090 (16)	0.54104 (19)	0.0461 (5)	
H28A	0.5337	0.1814	0.4897	0.055*	
H28B	0.6626	0.1328	0.4932	0.055*	
C29	0.67397 (18)	−0.01425 (14)	0.63682 (18)	0.0366 (4)	
C30	0.6074 (2)	−0.10035 (16)	0.6639 (2)	0.0470 (5)	
H30A	0.5378	−0.1260	0.5822	0.056*	
H30B	0.5634	−0.0798	0.7146	0.056*	
C31	0.7096 (3)	−0.17909 (17)	0.7372 (3)	0.0558 (6)	
H31A	0.7735	−0.1559	0.8230	0.067*	
H31B	0.6622	−0.2341	0.7480	0.067*	
C32	0.7866 (3)	−0.20990 (19)	0.6659 (3)	0.0644 (7)	
H32A	0.7241	−0.2391	0.5836	0.077*	
H32B	0.8543	−0.2576	0.7170	0.077*	
C33	0.8567 (2)	−0.1242 (2)	0.6411 (3)	0.0594 (6)	
H33A	0.9014	−0.1450	0.5912	0.071*	
H33B	0.9257	−0.0991	0.7235	0.071*	
C34	0.7545 (2)	−0.04497 (18)	0.5671 (2)	0.0503 (5)	
H34A	0.6920	−0.0678	0.4807	0.060*	
H34B	0.8027	0.0102	0.5579	0.060*	

### Atomic displacement parameters (Å^2^)

**Table:**

	*U*^11^	*U*^22^	*U*^33^	*U*^12^	*U*^13^	*U*^23^
O1	0.0574 (8)	0.0422 (8)	0.0368 (7)	0.0038 (7)	0.0184 (6)	−0.0055 (6)
O2	0.0457 (8)	0.0503 (9)	0.0531 (9)	0.0017 (7)	0.0093 (7)	−0.0022 (7)
O3	0.0559 (8)	0.0485 (9)	0.0580 (9)	−0.0039 (7)	0.0342 (7)	−0.0076 (7)
O4	0.0471 (8)	0.0576 (10)	0.0765 (11)	−0.0064 (8)	0.0374 (8)	−0.0121 (9)
O5	0.0531 (7)	0.0372 (7)	0.0417 (7)	−0.0100 (6)	0.0259 (6)	−0.0030 (6)
O6	0.0378 (7)	0.0359 (7)	0.0567 (9)	−0.0033 (5)	0.0252 (6)	−0.0010 (6)
O7	0.0428 (7)	0.0305 (7)	0.0361 (7)	0.0007 (5)	0.0109 (6)	−0.0003 (5)
O8	0.0398 (7)	0.0402 (8)	0.0405 (8)	0.0027 (6)	0.0068 (6)	−0.0029 (6)
C1	0.0425 (10)	0.0443 (11)	0.0419 (11)	0.0033 (9)	0.0167 (8)	−0.0069 (9)
C2	0.0565 (13)	0.0526 (14)	0.0706 (16)	0.0012 (11)	0.0358 (12)	−0.0052 (12)
C3	0.0844 (17)	0.0558 (15)	0.0697 (16)	0.0028 (14)	0.0466 (15)	0.0019 (13)
C4	0.0810 (17)	0.0470 (13)	0.0732 (17)	0.0114 (12)	0.0393 (14)	−0.0044 (12)
C5	0.0609 (14)	0.0569 (14)	0.0657 (15)	0.0082 (11)	0.0297 (12)	−0.0168 (12)
C6	0.0628 (13)	0.0508 (13)	0.0480 (12)	−0.0052 (11)	0.0317 (11)	−0.0145 (10)
C7	0.0448 (10)	0.0462 (11)	0.0457 (11)	0.0041 (9)	0.0138 (9)	−0.0080 (9)
C8	0.0419 (10)	0.0433 (11)	0.0440 (11)	0.0022 (8)	0.0204 (9)	−0.0023 (9)
C9	0.0634 (13)	0.0496 (13)	0.0433 (12)	0.0041 (11)	0.0153 (10)	0.0026 (10)
C10	0.0414 (10)	0.0476 (11)	0.0613 (13)	0.0006 (9)	0.0277 (10)	−0.0051 (10)
C11	0.0643 (14)	0.0541 (14)	0.0638 (14)	−0.0017 (11)	0.0432 (12)	−0.0046 (11)
C12	0.0419 (10)	0.0477 (12)	0.0524 (12)	−0.0039 (9)	0.0245 (9)	−0.0042 (10)
C13	0.0524 (13)	0.0555 (14)	0.0600 (14)	−0.0031 (11)	0.0131 (11)	−0.0059 (11)
C14	0.0867 (18)	0.0547 (15)	0.0515 (14)	−0.0116 (13)	0.0282 (13)	−0.0115 (12)
C15	0.0853 (17)	0.0449 (13)	0.0734 (17)	−0.0066 (13)	0.0405 (15)	−0.0127 (12)
C16	0.0620 (14)	0.0519 (14)	0.0736 (17)	0.0106 (12)	0.0262 (13)	−0.0016 (12)
C17	0.0630 (14)	0.0546 (13)	0.0470 (12)	0.0037 (11)	0.0213 (11)	0.0035 (11)
C18	0.0372 (9)	0.0320 (9)	0.0378 (9)	−0.0028 (7)	0.0165 (7)	0.0003 (7)
C19	0.0617 (13)	0.0437 (12)	0.0401 (11)	−0.0114 (10)	0.0105 (10)	0.0020 (9)
C20	0.0660 (14)	0.0536 (14)	0.0496 (13)	−0.0235 (12)	0.0078 (11)	−0.0037 (11)
C21	0.0843 (17)	0.0354 (11)	0.0634 (15)	−0.0142 (11)	0.0351 (13)	−0.0067 (10)
C22	0.0659 (13)	0.0351 (11)	0.0575 (14)	0.0025 (10)	0.0251 (11)	0.0080 (10)
C23	0.0407 (10)	0.0385 (10)	0.0508 (11)	0.0033 (8)	0.0181 (9)	−0.0012 (9)
C24	0.0359 (8)	0.0354 (9)	0.0475 (10)	−0.0046 (8)	0.0196 (8)	−0.0041 (8)
C25	0.0363 (8)	0.0320 (9)	0.0337 (9)	0.0022 (7)	0.0136 (7)	0.0021 (7)
C26	0.0497 (10)	0.0369 (10)	0.0476 (11)	0.0022 (9)	0.0285 (9)	0.0042 (9)
C27	0.0345 (9)	0.0329 (9)	0.0453 (11)	−0.0001 (7)	0.0096 (8)	−0.0014 (8)
C28	0.0529 (11)	0.0410 (11)	0.0342 (10)	0.0057 (9)	0.0119 (9)	0.0028 (8)
C29	0.0362 (8)	0.0347 (9)	0.0338 (9)	0.0012 (7)	0.0121 (7)	−0.0029 (8)
C30	0.0464 (10)	0.0383 (11)	0.0557 (12)	−0.0057 (9)	0.0236 (9)	−0.0058 (9)
C31	0.0662 (14)	0.0373 (11)	0.0626 (15)	0.0018 (10)	0.0291 (12)	0.0051 (10)
C32	0.0745 (16)	0.0414 (12)	0.0745 (17)	0.0155 (12)	0.0326 (13)	−0.0014 (12)
C33	0.0556 (12)	0.0603 (15)	0.0696 (15)	0.0125 (11)	0.0354 (11)	−0.0042 (12)
C34	0.0559 (12)	0.0496 (13)	0.0518 (12)	0.0006 (10)	0.0307 (10)	−0.0039 (10)

### Geometric parameters (Å, °)

**Table:**

O1—C7	1.417 (3)	C15—C16	1.521 (4)
O1—C1	1.424 (2)	C15—H15A	0.9700
O2—C9	1.424 (3)	C15—H15B	0.9700
O2—C1	1.428 (3)	C16—C17	1.514 (4)
O3—C10	1.409 (3)	C16—H16A	0.9700
O3—C12	1.430 (3)	C16—H16B	0.9700
O4—C12	1.414 (3)	C17—H17A	0.9700
O4—C11	1.426 (3)	C17—H17B	0.9700
O5—C24	1.421 (2)	C18—C19	1.513 (3)
O5—C18	1.423 (2)	C18—C23	1.528 (3)
O6—C26	1.423 (3)	C19—C20	1.516 (3)
O6—C18	1.426 (2)	C19—H19A	0.9700
O7—C29	1.423 (2)	C19—H19B	0.9700
O7—C27	1.427 (2)	C20—C21	1.515 (4)
O8—C28	1.418 (3)	C20—H20A	0.9700
O8—C29	1.429 (2)	C20—H20B	0.9700
C1—C2	1.509 (3)	C21—C22	1.519 (3)
C1—C6	1.529 (3)	C21—H21A	0.9700
C2—C3	1.518 (4)	C21—H21B	0.9700
C2—H2A	0.9700	C22—C23	1.525 (3)
C2—H2B	0.9700	C22—H22A	0.9700
C3—C4	1.524 (4)	C22—H22B	0.9700
C3—H3A	0.9700	C23—H23A	0.9700
C3—H3B	0.9700	C23—H23B	0.9700
C4—C5	1.508 (4)	C24—C25	1.525 (3)
C4—H4A	0.9700	C24—H24A	0.9700
C4—H4B	0.9700	C24—H24B	0.9700
C5—C6	1.511 (4)	C25—C26	1.522 (3)
C5—H5A	0.9700	C25—C28	1.525 (3)
C5—H5B	0.9700	C25—C27	1.530 (3)
C6—H6A	0.9700	C26—H26A	0.9700
C6—H6B	0.9700	C26—H26B	0.9700
C7—C8	1.528 (3)	C27—H27A	0.9700
C7—H7A	0.9700	C27—H27B	0.9700
C7—H7B	0.9700	C28—H28A	0.9700
C8—C11	1.517 (3)	C28—H28B	0.9700
C8—C9	1.522 (3)	C29—C30	1.517 (3)
C8—C10	1.528 (3)	C29—C34	1.526 (3)
C9—H9A	0.9700	C30—C31	1.520 (3)
C9—H9B	0.9700	C30—H30A	0.9700
C10—H10A	0.9700	C30—H30B	0.9700
C10—H10B	0.9700	C31—C32	1.509 (4)
C11—H11A	0.9700	C31—H31A	0.9700
C11—H11B	0.9700	C31—H31B	0.9700
C12—C13	1.515 (3)	C32—C33	1.523 (4)
C12—C17	1.528 (3)	C32—H32A	0.9700
C13—C14	1.519 (4)	C32—H32B	0.9700
C13—H13A	0.9700	C33—C34	1.527 (3)
C13—H13B	0.9700	C33—H33A	0.9700
C14—C15	1.504 (4)	C33—H33B	0.9700
C14—H14A	0.9700	C34—H34A	0.9700
C14—H14B	0.9700	C34—H34B	0.9700
			
C7—O1—C1	114.73 (17)	C12—C17—H17A	109.1
C9—O2—C1	113.24 (16)	C16—C17—H17B	109.1
C10—O3—C12	115.24 (17)	C12—C17—H17B	109.1
C12—O4—C11	114.35 (16)	H17A—C17—H17B	107.8
C24—O5—C18	114.58 (14)	O5—C18—O6	110.48 (15)
C26—O6—C18	114.70 (14)	O5—C18—C19	105.91 (15)
C29—O7—C27	114.47 (15)	O6—C18—C19	105.59 (16)
C28—O8—C29	113.29 (15)	O5—C18—C23	111.72 (15)
O1—C1—O2	109.17 (17)	O6—C18—C23	111.99 (16)
O1—C1—C2	106.11 (18)	C19—C18—C23	110.81 (17)
O2—C1—C2	106.56 (17)	C18—C19—C20	112.22 (19)
O1—C1—C6	112.50 (17)	C18—C19—H19A	109.2
O2—C1—C6	111.55 (18)	C20—C19—H19A	109.2
C2—C1—C6	110.64 (19)	C18—C19—H19B	109.2
C1—C2—C3	112.3 (2)	C20—C19—H19B	109.2
C1—C2—H2A	109.1	H19A—C19—H19B	107.9
C3—C2—H2A	109.1	C21—C20—C19	111.6 (2)
C1—C2—H2B	109.1	C21—C20—H20A	109.3
C3—C2—H2B	109.1	C19—C20—H20A	109.3
H2A—C2—H2B	107.9	C21—C20—H20B	109.3
C2—C3—C4	111.6 (2)	C19—C20—H20B	109.3
C2—C3—H3A	109.3	H20A—C20—H20B	108.0
C4—C3—H3A	109.3	C20—C21—C22	110.6 (2)
C2—C3—H3B	109.3	C20—C21—H21A	109.5
C4—C3—H3B	109.3	C22—C21—H21A	109.5
H3A—C3—H3B	108.0	C20—C21—H21B	109.5
C5—C4—C3	110.0 (2)	C22—C21—H21B	109.5
C5—C4—H4A	109.7	H21A—C21—H21B	108.1
C3—C4—H4A	109.7	C21—C22—C23	111.3 (2)
C5—C4—H4B	109.7	C21—C22—H22A	109.4
C3—C4—H4B	109.7	C23—C22—H22A	109.4
H4A—C4—H4B	108.2	C21—C22—H22B	109.4
C4—C5—C6	111.7 (2)	C23—C22—H22B	109.4
C4—C5—H5A	109.3	H22A—C22—H22B	108.0
C6—C5—H5A	109.3	C22—C23—C18	111.61 (17)
C4—C5—H5B	109.3	C22—C23—H23A	109.3
C6—C5—H5B	109.3	C18—C23—H23A	109.3
H5A—C5—H5B	107.9	C22—C23—H23B	109.3
C5—C6—C1	111.52 (19)	C18—C23—H23B	109.3
C5—C6—H6A	109.3	H23A—C23—H23B	108.0
C1—C6—H6A	109.3	O5—C24—C25	111.35 (15)
C5—C6—H6B	109.3	O5—C24—H24A	109.4
C1—C6—H6B	109.3	C25—C24—H24A	109.4
H6A—C6—H6B	108.0	O5—C24—H24B	109.4
O1—C7—C8	112.05 (17)	C25—C24—H24B	109.4
O1—C7—H7A	109.2	H24A—C24—H24B	108.0
C8—C7—H7A	109.2	C26—C25—C24	107.28 (16)
O1—C7—H7B	109.2	C26—C25—C28	111.19 (16)
C8—C7—H7B	109.2	C24—C25—C28	110.07 (16)
H7A—C7—H7B	107.9	C26—C25—C27	109.84 (15)
C11—C8—C9	111.17 (19)	C24—C25—C27	110.74 (16)
C11—C8—C10	107.25 (19)	C28—C25—C27	107.74 (16)
C9—C8—C10	109.61 (18)	O6—C26—C25	111.19 (16)
C11—C8—C7	109.71 (18)	O6—C26—H26A	109.4
C9—C8—C7	107.93 (18)	C25—C26—H26A	109.4
C10—C8—C7	111.18 (19)	O6—C26—H26B	109.4
O2—C9—C8	111.81 (19)	C25—C26—H26B	109.4
O2—C9—H9A	109.3	H26A—C26—H26B	108.0
C8—C9—H9A	109.3	O7—C27—C25	111.24 (15)
O2—C9—H9B	109.3	O7—C27—H27A	109.4
C8—C9—H9B	109.3	C25—C27—H27A	109.4
H9A—C9—H9B	107.9	O7—C27—H27B	109.4
O3—C10—C8	112.26 (16)	C25—C27—H27B	109.4
O3—C10—H10A	109.2	H27A—C27—H27B	108.0
C8—C10—H10A	109.2	O8—C28—C25	111.70 (16)
O3—C10—H10B	109.2	O8—C28—H28A	109.3
C8—C10—H10B	109.2	C25—C28—H28A	109.3
H10A—C10—H10B	107.9	O8—C28—H28B	109.3
O4—C11—C8	110.98 (18)	C25—C28—H28B	109.3
O4—C11—H11A	109.4	H28A—C28—H28B	107.9
C8—C11—H11A	109.4	O7—C29—O8	109.78 (15)
O4—C11—H11B	109.4	O7—C29—C30	105.44 (16)
C8—C11—H11B	109.4	O8—C29—C30	105.98 (15)
H11A—C11—H11B	108.0	O7—C29—C34	112.77 (15)
O4—C12—O3	110.06 (18)	O8—C29—C34	111.65 (17)
O4—C12—C13	106.08 (18)	C30—C29—C34	110.83 (17)
O3—C12—C13	104.72 (19)	C29—C30—C31	112.07 (17)
O4—C12—C17	111.48 (19)	C29—C30—H30A	109.2
O3—C12—C17	113.44 (18)	C31—C30—H30A	109.2
C13—C12—C17	110.6 (2)	C29—C30—H30B	109.2
C12—C13—C14	112.3 (2)	C31—C30—H30B	109.2
C12—C13—H13A	109.2	H30A—C30—H30B	107.9
C14—C13—H13A	109.2	C32—C31—C30	111.3 (2)
C12—C13—H13B	109.2	C32—C31—H31A	109.4
C14—C13—H13B	109.2	C30—C31—H31A	109.4
H13A—C13—H13B	107.9	C32—C31—H31B	109.4
C15—C14—C13	112.1 (2)	C30—C31—H31B	109.4
C15—C14—H14A	109.2	H31A—C31—H31B	108.0
C13—C14—H14A	109.2	C31—C32—C33	110.8 (2)
C15—C14—H14B	109.2	C31—C32—H32A	109.5
C13—C14—H14B	109.2	C33—C32—H32A	109.5
H14A—C14—H14B	107.9	C31—C32—H32B	109.5
C14—C15—C16	110.4 (2)	C33—C32—H32B	109.5
C14—C15—H15A	109.6	H32A—C32—H32B	108.1
C16—C15—H15A	109.6	C32—C33—C34	111.14 (19)
C14—C15—H15B	109.6	C32—C33—H33A	109.4
C16—C15—H15B	109.6	C34—C33—H33A	109.4
H15A—C15—H15B	108.1	C32—C33—H33B	109.4
C17—C16—C15	112.0 (2)	C34—C33—H33B	109.4
C17—C16—H16A	109.2	H33A—C33—H33B	108.0
C15—C16—H16A	109.2	C33—C34—C29	111.66 (19)
C17—C16—H16B	109.2	C33—C34—H34A	109.3
C15—C16—H16B	109.2	C29—C34—H34A	109.3
H16A—C16—H16B	107.9	C33—C34—H34B	109.3
C16—C17—C12	112.6 (2)	C29—C34—H34B	109.3
C16—C17—H17A	109.1	H34A—C34—H34B	107.9
			
C7—O1—C1—O2	57.4 (2)	C24—O5—C18—O6	−54.30 (19)
C7—O1—C1—C2	171.85 (17)	C24—O5—C18—C19	−168.18 (17)
C7—O1—C1—C6	−67.0 (2)	C24—O5—C18—C23	71.1 (2)
C9—O2—C1—O1	−58.4 (2)	C26—O6—C18—O5	54.3 (2)
C9—O2—C1—C2	−172.63 (18)	C26—O6—C18—C19	168.41 (16)
C9—O2—C1—C6	66.5 (2)	C26—O6—C18—C23	−70.9 (2)
O1—C1—C2—C3	68.8 (2)	O5—C18—C19—C20	−175.2 (2)
O2—C1—C2—C3	−174.9 (2)	O6—C18—C19—C20	67.6 (2)
C6—C1—C2—C3	−53.5 (3)	C23—C18—C19—C20	−53.9 (3)
C1—C2—C3—C4	54.9 (3)	C18—C19—C20—C21	55.4 (3)
C2—C3—C4—C5	−55.4 (3)	C19—C20—C21—C22	−55.8 (3)
C3—C4—C5—C6	56.6 (3)	C20—C21—C22—C23	56.0 (3)
C4—C5—C6—C1	−56.6 (3)	C21—C22—C23—C18	−55.5 (3)
O1—C1—C6—C5	−64.3 (3)	O5—C18—C23—C22	171.80 (17)
O2—C1—C6—C5	172.62 (18)	O6—C18—C23—C22	−63.7 (2)
C2—C1—C6—C5	54.2 (3)	C19—C18—C23—C22	54.0 (2)
C1—O1—C7—C8	−54.7 (2)	C18—O5—C24—C25	55.9 (2)
O1—C7—C8—C11	170.23 (17)	O5—C24—C25—C26	−53.1 (2)
O1—C7—C8—C9	49.0 (2)	O5—C24—C25—C28	−174.18 (15)
O1—C7—C8—C10	−71.3 (2)	O5—C24—C25—C27	66.8 (2)
C1—O2—C9—C8	57.6 (2)	C18—O6—C26—C25	−55.9 (2)
C11—C8—C9—O2	−171.00 (18)	C24—C25—C26—O6	52.9 (2)
C10—C8—C9—O2	70.6 (2)	C28—C25—C26—O6	173.35 (16)
C7—C8—C9—O2	−50.6 (2)	C27—C25—C26—O6	−67.5 (2)
C12—O3—C10—C8	−53.6 (3)	C29—O7—C27—C25	55.4 (2)
C11—C8—C10—O3	51.2 (2)	C26—C25—C27—O7	−171.77 (16)
C9—C8—C10—O3	171.96 (19)	C24—C25—C27—O7	69.9 (2)
C7—C8—C10—O3	−68.8 (2)	C28—C25—C27—O7	−50.5 (2)
C12—O4—C11—C8	57.9 (3)	C29—O8—C28—C25	−57.5 (2)
C9—C8—C11—O4	−172.61 (18)	C26—C25—C28—O8	172.47 (16)
C10—C8—C11—O4	−52.8 (2)	C24—C25—C28—O8	−68.8 (2)
C7—C8—C11—O4	68.1 (2)	C27—C25—C28—O8	52.1 (2)
C11—O4—C12—O3	−55.6 (2)	C27—O7—C29—O8	−57.0 (2)
C11—O4—C12—C13	−168.4 (2)	C27—O7—C29—C30	−170.75 (15)
C11—O4—C12—C17	71.1 (3)	C27—O7—C29—C34	68.2 (2)
C10—O3—C12—O4	53.6 (2)	C28—O8—C29—O7	57.7 (2)
C10—O3—C12—C13	167.21 (18)	C28—O8—C29—C30	171.09 (16)
C10—O3—C12—C17	−72.1 (2)	C28—O8—C29—C34	−68.1 (2)
O4—C12—C13—C14	−173.8 (2)	O7—C29—C30—C31	−68.2 (2)
O3—C12—C13—C14	69.8 (3)	O8—C29—C30—C31	175.39 (18)
C17—C12—C13—C14	−52.8 (3)	C34—C29—C30—C31	54.1 (2)
C12—C13—C14—C15	55.6 (3)	C29—C30—C31—C32	−55.7 (3)
C13—C14—C15—C16	−55.5 (3)	C30—C31—C32—C33	56.2 (3)
C14—C15—C16—C17	54.8 (3)	C31—C32—C33—C34	−56.1 (3)
C15—C16—C17—C12	−53.9 (3)	C32—C33—C34—C29	55.2 (3)
O4—C12—C17—C16	170.1 (2)	O7—C29—C34—C33	64.1 (2)
O3—C12—C17—C16	−65.0 (3)	O8—C29—C34—C33	−171.77 (18)
C13—C12—C17—C16	52.3 (3)	C30—C29—C34—C33	−53.9 (2)
